# PIBAS FedSPARQL: a web-based platform for integration and exploration of bioinformatics datasets

**DOI:** 10.1186/s13326-017-0151-z

**Published:** 2017-09-20

**Authors:** Marija Djokic-Petrovic, Vladimir Cvjetkovic, Jeremy Yang, Marko Zivanovic, David J. Wild

**Affiliations:** 1 Virtual World Services GmbH, Asperner Heldenplatz 6, 1220 Wien, Austria; 2 Department of Mathematics and Informatics, Faculty of Science, University of Kragujevac, Radoja Domanovica 12, Kragujevac, 34000 Serbia; 3 School of Informatics and Computing, Indiana University, 901 E 10th St, Bloomington, Indiana, 47408 USA; 4 Translational Informatics Division, School of Medicine, University of New Mexico, Albuquerque, NM 87131 USA; 5 Department of Biology and Ecology, Faculty of Science, University of Kragujevac, Radoja Domanovica 12, Kragujevac, 34 000 Serbia

**Keywords:** Federated SPARQL query, Bioinformatics, Data integration, Ontologies, Data mining and information retrieval

## Abstract

**Background:**

There are a huge variety of data sources relevant to chemical, biological and pharmacological research, but these data sources are highly siloed and cannot be queried together in a straightforward way. Semantic technologies offer the ability to create links and mappings across datasets and manage them as a single, linked network so that searching can be carried out across datasets, independently of the source. We have developed an application called PIBAS FedSPARQL that uses semantic technologies to allow researchers to carry out such searching across a vast array of data sources.

**Results:**

PIBAS FedSPARQL is a web-based query builder and result set visualizer of bioinformatics data. As an advanced feature, our system can detect similar data items identified by different Uniform Resource Identifiers (URIs), using a text-mining algorithm based on the processing of named entities to be used in Vector Space Model and Cosine Similarity Measures. According to our knowledge, PIBAS FedSPARQL was unique among the systems that we found in that it allows detecting of similar data items. As a query builder, our system allows researchers to intuitively construct and run Federated SPARQL queries across multiple data sources, including global initiatives, such as Bio2RDF, Chem2Bio2RDF, EMBL-EBI, and one local initiative called CPCTAS, as well as additional user-specified data source. From the input topic, subtopic, template and keyword, a corresponding initial Federated SPARQL query is created and executed. Based on the data obtained, end users have the ability to choose the most appropriate data sources in their area of interest and exploit their Resource Description Framework (RDF) structure, which allows users to select certain properties of data to enhance query results.

**Conclusions:**

The developed system is flexible and allows intuitive creation and execution of queries for an extensive range of bioinformatics topics. Also, the novel “similar data items detection” algorithm can be particularly useful for suggesting new data sources and cost optimization for new experiments. PIBAS FedSPARQL can be expanded with new topics, subtopics and templates on demand, rendering information retrieval more robust.

## Background

### Motivation

Nowadays, large amounts of bioinformatics data are publicly available to researchers of the life science community. These data and associated annotations are accessible through heterogeneous databases hosted as part of many independent and highly specialized resources and represented in different formats, conventions, vocabularies and ontologies. Still, modern research in bioinformatics greatly depends on the availability and efficient use of these data. Scientific research often requires access to various data points across scattered and highly distributed sources. This makes finding relevant data for scientific research projects a difficult and laborious task. With the rapid accumulation of bioinformatics data, this issue has only become more important and challenging.

The lack of integrated solutions that would contribute to better results and discovering of new knowledge is a primary issue in the bioinformatics community [1]. Hence, the bioinformatics community has increasingly taken to employing Semantic Web technologies for better and easier data integration. The benefits of this approach include aggregation of heterogeneous data using explicit semantics, and simplified annotation and expression of rich and well-defined models for data aggregation and searching [2]. Therefore, the grand vision and practical technologies of the Semantic Web offer a possibility of solving longstanding problems of data integration in bioinformatics [3].

Motivated and influenced by the ongoing needs of supporting the research activities of the PIBAS (CPCTAS-LCMB) Research Center (RC) [4], the authors have successfully employed Semantic Web technologies, enabling integration of external and internal bioinformatics datasets. RC is a laboratory for testing bioactive substances which are candidates for use in pharmaceutical therapeutics. Work at RC includes monitoring of in vitro effects of active substances in cell lines of different origin (primarily cancer cell lines) and primary cells isolated from other types of tissue. Experiments carried out in RC include measuring the effectiveness of a substance in inhibiting a specific biological function (IC_50_) in human cancer cell lines and quantifying the mechanisms of apoptosis, migration and angiogenesis. The experimental data obtained at RC are varied and complex and represent intertwined relationships among various terms and concepts used at RC. This complex data structure is represented as an ontology [5]. The ontology simplifies the search for experimental data and comprises a formal, rigorous representation of the conceptual model of the domain.

The main subjects that RC staff are interested in are information about targets, bioassays and cell lines used in earlier experiments. In addition to the PIBAS ontology [5], which provides internal support to RC staff, supplementary information can be extracted from global initiatives such as Bio2RDF [6], Chem2Bio2RDF [7] and the EMBL-EBI platform [8]. For example, information about targets can be found in ChEMBL [9], BindingDB [10] and Drugbank [11] datasets, form the EMBL-EBI, Chem2Bio2RDF and Bio2RDF initiatives, respectively. The necessary information for bioassays can be found in ChEMBL and Pubchem [12] datasets form the EMBL-EBI and Chem2Bio2RDF initiatives, respectively. Information about cell lines can be found in ChEMBL and ChemBank [13] datasets from the EMBL-EBI and Chem2Bio2RDF initiatives, respectively. Another search requirement is investigation of actual research results in publications. For example, information about publications can be found via PubMed [14], from the Bio2RDF initiative, as well as in the local Reference ontology [15] developed for internal use at RC. In previous work [16], the authors focused on integration of these initiatives. Based on manually entered data, such as InChi, InChiKey, SMILES or molecular formula, the system offers templates and generates static Federated SPARQL queries [17] for retrieval of relevant information. This system has been very helpful in discovering new knowledge, but in the light of ever-increasing volume of experimental data, the needs of RC mandated the development of a new system. One of the main requirements in this regard was the inclusion of relevant and new datasets in predefined queries to make it possible to find complementary information about data items (targets, bioassays and cell lines). An additional requirement was the capability to detect similar data items to increase the performance of experiments and lower processing costs. This is one of the major challenges in the bioinformatics community, as the data items are represented by distinct URIs at different endpoints [18], which necessitated a serious effort to discover and compare their common properties.

In order to meet the above-mentioned requirements of RC, the authors developed PIBAS FedSPARQL,^1^ a platform based on Semantic Web technologies that allows end users to easily provide input data and run predefined Federated SPARQL queries across multiple data sources and detect similar data items, among data obtained from a query. For the process of detecting similar data items, the authors developed a text-mining algorithm based on the processing of object values (strings) of the named entities to be used in Vector Space Model (VSM) [19] and Cosine Similarity Measures (CSM) [20]. Also, one of the features of PIBAS FedSPARQL is the capability of filtering results obtained by a query. Filtering is based on a projection of RDF data sources included in the query. Searching and sorting of results is also offered. Users can add additional data source if they are interested in querying endpoint that is not contained in the predefined query. The system can also be extended with new topics, subtopics and templates on demand.

### Features

Adhering to the philosophy of Arsic et al. [16], the authors implemented the following SPARQL features:

- Federation: Federated SPARQL queries over remote endpoints, gather novel and complementary data about targets, bioassays and cell lines in real time. This eliminates constant update monitoring.
- Scalability: Data integration with user-specified data sources is possible. Furthermore, end users have the ability to choose the most appropriate data sources in their area of interest and exploit their RDF structure. This allows them to select certain properties of data sources to improve query results.
- Advancement: Detecting similar data items using a method based on text-mining. This feature is helpful for optimizing the costs of new experiments.
- Availability: Locally used RC data are now public and available to the entire bioinformatics community.

The rest of the paper is organized as follows: The next subsection represents a survey on related works. In the Implementation section, we present the architecture of PIBAS FedSPARQL. In the Methods section, we describe all features of PIBAS FedSPARQL and highlight our algorithm for similar data items detection, explaining it in detail and presenting a use case. In the Results section we present the results obtained through an evaluation. In the Conclusions and future work section, apart from presenting the final remarks, we also outline a possible approach for future work. The section Appendices contains various definitions used in our study.

### Related work

In modern biology and chemistry, exploiting the diverse kinds of available data about a topic of interest is challenging, as data are spread over many sources. Bioinformatics datasets are highly distributed and heterogeneous, and this heterogeneity exists at many levels including data formats, conventions and meaning. Due to these factors, traditional approaches for data searching often deliver unsatisfactory results. The need for an integrated solution has led many organizations to use the Semantic Web, because of its wide range of possibilities. The Semantic Web is recognized as a common framework that allows data to be used and shared across applications and database boundaries [21].

Initiatives such as Bio2RDF [6] and LODD [22] address the problem of connecting biological and drug data. Bio2RDF has transfigured and interrelated many biological databases, offering a platform for constructing queries across these data sources. The LODD initiative integrates various sources of drug data, motivated by domain-aware scientific questions. Chem2Bio2RDF [7] aggregates data from various data sources that are contained in Bio2RDF and LODD. It covers around 25 distinct datasets with connected compounds, drugs, pathways, side effects, genes, diseases and PubMed documents. Chem2Bio2RDF also includes a tool to facilitate queries and a set of comprehensive functions to address specific research requests. EMBL-EBI [8] contains a wide range of freely accessible molecular data sources, such as UniProt [23], ChEMBL and Reactome [24]. Open PHACTS [25] is a unique initiative developed as a shared platform for integration and knowledge discovery. It constitutes an approach based on the Semantic Web to address bottlenecks in drug discovery. The project mainly focuses on distinct information sources, lack of standards and information overcharge as major issues. Its goals are establishing open standards and creating infrastructure for research cooperation. Projects such as LinkHub [26], SWIT [27] and BioGateway [28] also offer their solutions for the integration of bioinformatics data.

All the solutions mentioned above have many datasets in common and together they combine vast amounts of bioinformatics data. Besides profound background knowledge about the underlying data sources, users also need to have solid command of the SPARQL query language to successfully access the data. SPARQL is an RDF query language used to retrieve and control data stored in RDF graphs [29]. SPARQL also allows executing queries that are distributed over multiple endpoints, so-called Federated SPARQL queries [30]. Generally, SPARQL has a complex syntax that is difficult to work with for inexperienced users and, consequently, querying data is a problem for many researchers. Therefore, a number of existing applications strive to provide a user-friendly interface for browsing bioinformatics data or to allow users to perform Federated SPARQL queries. Several of these solutions are described below.

SPARQLGraph [31] is a web-based platform for the visual creation and execution of biological SPARQL queries. The graphical query builder allows end users to create and share query graphs in a simple way. Several template queries are provided, offering a great starting point for building new graphs and assisting researchers in finding answers to biological questions. In the SPARQLGraph the datasets are integrated in the interface internally and no other datasets are supported. In PIBAS FedSPARQL some datasets are integrated and end users can also add an outside dataset if they want to query endpoints that are not in the list of integrated datasets. Both interfaces provide template queries in multiple datasets and enable end users to choose from these datasets to facilitate direct querying.

QueryMed [18] allows queries relevant to a wide range of biomedical topics. It runs federated queries across multiple SPARQL endpoints. QueryMed is designed to be accessible to users who are not familiar with the underlying ontologies or the SPARQL query language. The system allows users to select the data sources they wish to use. Users can also add additional data sources. After retrieval of the initial result set, query results can be filtered to improve their relevance. As an advanced search feature, the system also allows users to exploit the underlying structure of the RDF data to improve query results. This solution is the most similar to our approach, but the main difference lies in the fact that PIBAS FedSPARQL offers a feature for finding similar data items in the retrieved result set.

Twinkle [32] provides a stand-alone graphical user interface to load and edit SPARQL queries. In this case, users are expected to know what is already available at the SPARQL endpoints and to write the queries that can be used to directly query remote SPARQL endpoints. This approach is the opposite of ours: initial PIBAS FedSPARQL queries are predefined, while conversance of SPARQL is necessary for adding new datasets. Although Twinkle was mostly designed as a general purpose system, it only supports a small number of specific SPARQL endpoints, while PIBAS FedSPARQL allows users to add any new SPARQL endpoint.

GoWeb [33] was created for answering queries on biomedical data. It lets users run old-style keyword-based web searches with ontology search features. After a keyword search, documents can be filtered based on the biomedical annotations they contain. Nevertheless, in GoWeb the exact queried sources are not transparent and cannot be selected or customized by end users as in PIBAS FedSPARQL.

The SMART [34] query tool is a web-based application that allows biology researchers to run SPARQL queries over multiple data sources. Their queries are constructed using a description logic written in the Manchester OWL syntax [35]. In contrast, PIBAS FedSPARQL allows end users to intuitively run predefined queries by selecting topics, subtopics, templates and entering keywords without requiring background knowledge about the SPARQL syntax.

BioQueries [36] lets users to design and share SPARQL queries that can simplify and reduce many common and frequent bioinformatics data retrieval tasks. The BioQueries interface provides context-specific anchoring for queries via the use of placeholders. Queries are represented as a sentence with one or more gaps where a user can enter context-specific information. In the PIBAS FedSPARQL system, Federated SPARQL queries are displayed as a corresponding virtual sentence based on the items selected and keyword entered.

FedX [37] runs queries over either Sesame repositories^2^ or SPARQL endpoints. During the initial phase, it loads the list of data sources without its statistical information. The source selection is done by sending SPARQL ASK queries. The size of intermediate result is minimized by a rule-based join optimizer according to a cost estimation. By contrast, PIBAS FedSPARQL preserves intermediate results because it is very important for RC staff to gain all relevant data.

To overcome the problem of querying multiple data sources, which can vary in their RDF representations, proficiency in SPARQL is essential, but usually not sufficient, for successful information retrieval from such data sources. Identifying relevant data sources and discovering their capabilities and the type of data they contain is a process known as source discovery [38] and a necessary pre-step for determining whether a particular data source matches researchers’ demands. There are often many alternative ways of carrying out source discovery [38], all of varying efficiency, and SPARQL experts have to choose from these options. Our approach for solving these challenges is based on close co-operation with RC experts. In order to fulfill the requirements of RC, we carried out a source discovery process and arrived at Bio2RDF, Chem2Bio2RDF and EMBL-EBI as viable data sources (initiatives). Then, a series of small SPARQL queries were created from pattern queries that were partly handpicked from initiative examples and handcrafted. Furthermore, we interoperated between data sources, tracking and linking related instances, which we received as results from executing the series of the SPARQL queries. Assessing the results, we picked up suitable handcrafted pattern queries and created the final SPARQL queries for each requirement. Thus, PIBAS FedSPARQL federates data by executing already predefined Federated SPARQL queries and this is different from a federated query engine BioFed [39] that is able to federate more than 130 public SPARQL endpoints. In BioFed queries are built based on existing data and then distributed to the relevant endpoints through a source selection approach.

Although integrated approaches in the bioinformatics domain are available, there are still a number of challenges that must be addressed in order to make such resources accessible to researchers. Data warehousing within bioinformatics information infrastructures in order to enable semantic interoperability between its various stakeholders, is one of the main challenges [40]. A simple form of a data warehouse that is focused on a single subject is called a data mart [41]. Depending on the requirements and complexity of the system, there are several types of implementation of data warehousing. For example, Open PHACTS [25] uses a bottom-up approach, where the data marts are created first and then combined into a single, all-encompassing data warehouse. Generally, in data management, semantic warehousing is a methodology of digitalizing text data using similar functions as data warehousing such as ETL (extract, transform, load) [40]. In PIBAS FedSPARQL authors do not use semantic warehousing, although the VSM approach employed can be seen as a data mart solution in the sense that extracted semantic information (text) is transformed and prepared for usage in CSM.

One of the most intriguing problems in the bioinformatics community is finding similar data items across the same or different initiatives [18]. PIBAS FedSPARQL offers a flexible and interesting way to overcome this challenge using a method based on text-mining. We apply VSM on terms, which are actually words or phrases from biological or chemical areas, and then compare the vectors using CSM. This algorithm is described in detail in the Methods section.

The study of semantic similarity between words has long been an integral part of information retrieval, natural language processing and the Semantic Web. Semantic similarity between entities changes over time and across domains. The rest of this paragraph outlines some traditional approaches to identifying semantic similarity. Given a taxonomy of concepts, a straightforward method to calculate similarity between two words (concepts) is to find the length of the shortest path connecting the two words in the taxonomy [42]. If a word is polysemous, then multiple paths might exist between the two words. In such cases, only the shortest path between any two senses of the words is considered for calculating similarity. A problem that is frequently acknowledged in relation to this approach is that it relies on the notion that all links in the taxonomy represent a uniform distance. Resnik [43] proposed a similarity measure using information content. This approach defines the similarity between two concepts C1 and C2 in the taxonomy as the maximum of the information content of all concepts C that subsume both C1 and C2. The similarity between two words, then, is defined as the maximum of the similarity between any concepts that the words belong to. Resnik used WordNet [44] as taxonomy and calculated information content using the Brown corpus [45]. Matsuo et al. [46] used a similar approach to measure the similarity between words and apply their method in a graph-based word-clustering algorithm.

Semantic similarity measures have been used in many Semantic Web applications. Ehrig et al. [47] describes a framework that aims at comparing concepts across ontologies, and not ontologies themselves. This is similar to our solution, where we only compare object values (concepts). David et al. [48] present a number of measures for ontology matching and state that simple measures like Cosine Similarity on a term-frequency vector give accurate results. This is also the measure method we use in our system.

In our previous work, we demonstrated the power of ontology-based information system [5]. A new ontology was developed for RC that contains encoded knowledge about local experimental structure and an ontological database was created that contains data from individual experiments. Additionally, to make it possible to find relevant information essential for the further performance of local experiments, a local approach for running static Federated SPARQL queries over CPCTAS [5], Bio2RDF, Chem2Bio2RDF and EMBL-EBI was created [16]. Currently, RC wanted to expand the search and discover complementary data by adding new dataset and finding similar data items to potentially narrow down the choice of materials and methods for future experiments. In this paper, the PIBAS FedSPARQL system is described, which implements these ontological, database and strategic approaches.

## Implementation

### Architecture overview

**Fig. 1:**
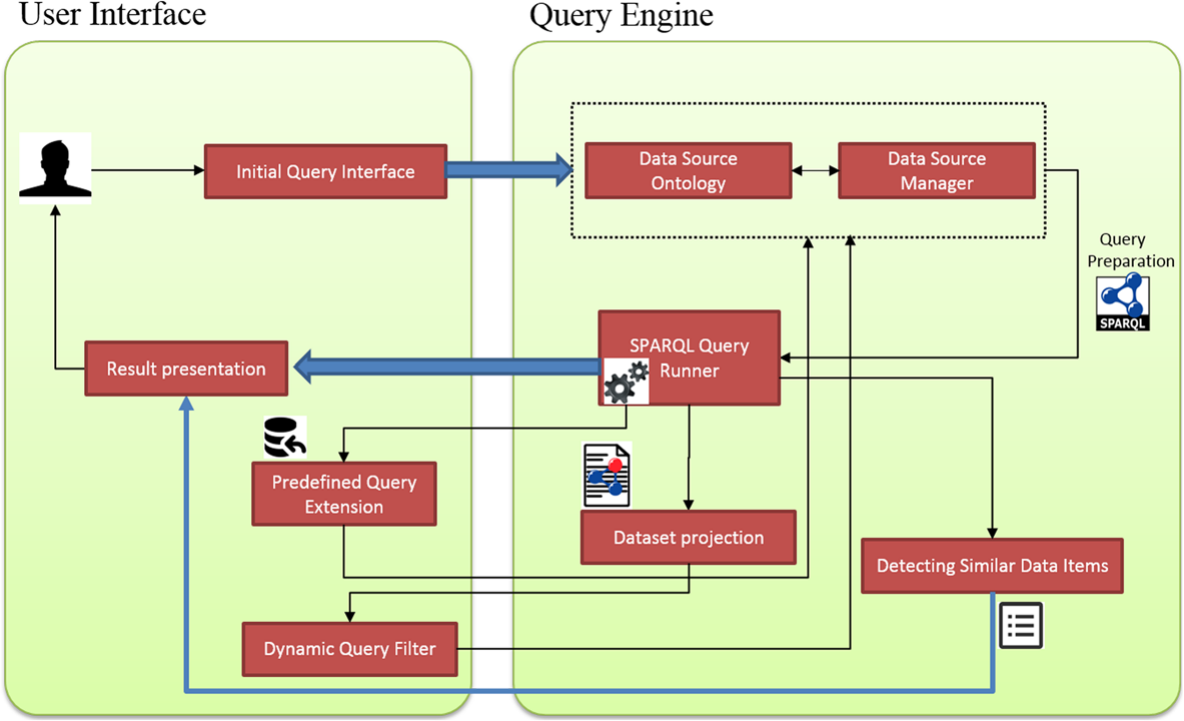
PIBAS FedSPARQL architecture overview. The architecture consists of two main layers: query engine and user interface. The user interface enables users to construct simple and advanced queries and view the results of their execution. The query engine preforms a series of demanding processes that needs to be done before queries can be executed. The main query engine component, *Data Source Manager*, scans the local *DataSources* ontology, reads the user’s input and passes the information through the *Query preparation* component to the SPARQL query runner component, where the queries are executed. The *Dataset projection* component plays a role in the “*Dynamic query filter*” feature, allowing users to easily discover the structure of underlying datasets included in Federated SPARQL queries. The *Detecting Similar Data Items* component identifies similar data items from results retrieved after running predefined queries or queries extended with new datasets

The PIBAS FedSPARQL architecture is shown in Fig. 1. The main components are user interface and query engine. The user interface enables users to construct simple and advanced queries and view the results of their execution, while the query engine executes queries across remote SPARQL endpoints. PIBAS FedSPARQL was implemented in PHP and Python. The JQuery library^3^ was used to develop an interactive and user-friendly interface, while sparqllib^4^ was used to run Federated SPARQL queries. The list of available datasets used for creating predefined Federated SPARQL queries is placed in the local *DataSources* ontology [49] developed using Protégé 4.0.2 [50].

### User interface

The user query interface was implemented in HTML, JQuery and JavaScript. Its core components are:

#### Initial query interface

Users can choose from predefined topics, subtopics and templates. The selection of subtopics is limited by of the topic selected. This also applies to the relation between topics and templates. All relations reflect the needs of the researchers at RC. Every template is based on a form of an underlying predefined Federated SPARQL query.

#### Predefined query extension

This component allows end users to add new datasets to the predefined Federated SPARQL queries.

#### Dynamic query filter

This component allows end users to select the desired datasets, load the properties available for these datasets and dynamically expand Federated SPARQL queries with selected properties.

#### Result presentation

This component allows end users to view the results of predefined queries in table form. One column shows retrieved results as URI or string, while another column displays data source and initiative name. End users can also apply a dynamic query filter to view the results organized by source. In both cases, the columns can be sorted and searched based on entered text.

### Query engine

PIBAS FedSPARQL runs Federated SPARQL queries on our local JOSEKI endpoint.^5^ Before the queries can be executed, a series of demanding processes need to be performed. These tasks are carried out by the following components:

#### Data source ontology

This component implies the *DataSources* ontology that contains the patterns of predefined queries for all templates as well as information about datasets that are initially included in queries.

#### Data source manager

This component scans the data source ontology and uses the corresponding datasets information to fulfill the user requirements. The data source manager also keeps track of predefined datasets and the datasets included in extended queries.

#### Dataset projection

This component returns properties for every dataset included in Federated SPARQL queries. End users can choose from a number of properties based on their description.

#### Query preparation

This component is in charge of translating and preparing the requirements of end users into valid Federated SPARQL queries. Requirements include selecting options from the initial query interface, adding new endpoints to predefined queries and dynamic query filtering.

#### SPARQL query runner

This component executes Federated SPARQL queries.

#### Detecting similar data items

This component detects similar data items (URIs) from results retrieved after running predefined queries or queries extended with new datasets. Similar data items are shown on a new web page.

## Methods

### Running of predefined queries

**Fig. 2:**
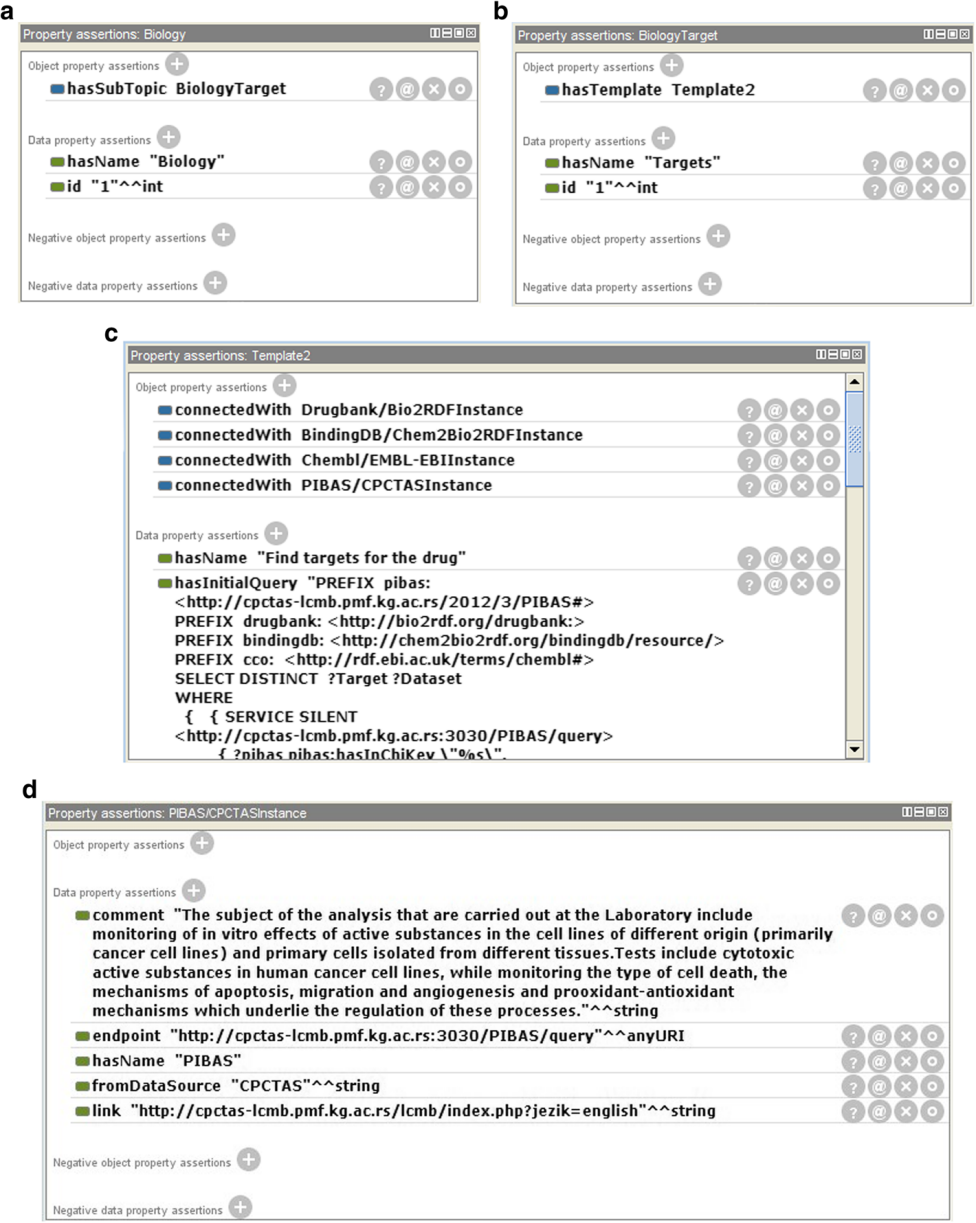
Representations of basic relations in the *DataSources* ontology in the Protégé editor **a**) *Topic Biology* **b**) *Subtopic BiologyTarget* **c**) Template “*Found targets for the drug*” and some of its properties **d**) *PIBAS/CPCTAS* dataset instance. This figure shows screenshots of the local ontology *DataSources* in the Protégé ontology editor [50]. The ontology contains information about initiatives and datasets included in predefined Federated SPARQL queries. Each dataset in the ontology is represented as an instance of a certain class. The object property *conectedWith* connects dataset instances with template instances. Every *Subtopic class* instance is connected with a *Template class* instance through the object property *hasTemplate*. Every *Topic class* instance is connected with a *Subtopic class* instance through the object property *hasSubTopic*

Information about initiatives and datasets included in predefined queries is placed in the local ontology *DataSources*. Each dataset is represented as an instance, while each template is connected to a dataset instance using the object property *connectedWith*. With respect to their purpose, the same dataset can be associated with a variety of templates. Every template belongs to a corresponding subtopic. Each subtopic has its own topic. For example, the topic *Biology* has the subtopic *BiologyTarget* while it is connected to *Template2* (see Fig. 2). *Template2* is created based on the following preselected datasets: *PIBAS/CPCTAS*, *BindingDB/Chem2Bio2RDF, Drugbank/Bio2RDF* and *ChEMBL/EMBL-EBI*.

Currently, the *DataSources* ontology contains topics that are created in accordance with the requirements of RC experts. Topics are divided into three areas: *Biology*, *Chemogenomic* and *Research*. All (sub)topics and templates are changeable and can easily be modified or added to. Templates can be modified in various ways. For example, the template *Find targets for the drug*, which requires the InChiKey value, can be transformed into a template that requires another value, such as SMILES. This change necessitates a manual modification in the predefined query. Templates can be expanded with one or more new datasets. Similarly, datasets can also be excluded from templates. A representation of all topics and their relations in PIBAS FedSPARQL is shown in Table 1.

**Table 1:** Representation of current (sub)topics and templates in the *DataSources* ontology

Topic	Subtopic	Template/Template label^*^	Keyword
Biology	Targets	Find targets for the drug/1	InChiKey
Chemogenomic	Assays	• Find assays for the drug/2	SMILE
	Cell lines	• Find cell lines for the drug/3	InChiKey
Research	Papers	Find papers with a title for the keyword/4	No restriction

^a^Template labels are used in Table 2 and Table 6

**Fig. 3:**
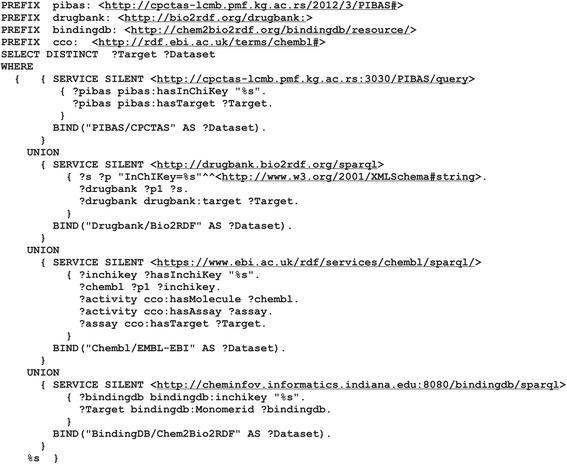
Predefined query of *Template2* for its pre-selected datasets. This figure shows the predefined Federated SPARQL query of the template “*Find targets for the drug*”. This query covers the *PIBAS/CPTAS*, *Drugbank/Bio2RDF*, *ChEMBL/EMBL-EBI* and *BindingDB/Chem2Bio2RDF* datasets. All predefined Federated SPARQL queries in the local *DataSources* ontology contain “%s” characters which represent objects values that will be replaced with the keyword entered by the user. The last “%s” character will be replaced with a particular pattern query if a new dataset is added using the “*Add new dataset*” feature

The property *hasInitialQuery* of each template represents a predefined Federated SPARQL query that runs across preselected datasets. Pattern queries for every dataset are collected from initiative examples and parts of them are handcrafted. Figure 3 shows the predefined query of *Template2*. All “%s” characters that represent objects in the predefined query will be replaced with the keyword entered by the end user, while the aftermost character is reserved for an additional dataset.

At the moment, PIBAS FedSPARQL uses datasets (Table 2) from the EMBL-EBI, Bio2RDF and Chem2Bio2RDF platforms. These are, used to establish the predefined Federated SPARQL queries. CPCTAS, as union of the PIBAS and Reference dataset, covers all the mentioned topics currently used for templates. Seeking to meet the needs of RC staff and highlight the importance of small laboratories, we have related the PIBAS dataset with templates from the *Biology* and *Chemogenomic* topics. The Reference dataset, as collection of ontologies, which describes references of scientific and research PMF^6^ staff, covers the *Research* topic.

**Table 2:** List of RDF datasets integrated in PIBAS FedSPARQL

PIBAS FedSPARQL			
Data source	Triples	Template label	Reference or dataset link
CPCTAS			
PIBAS dataset	437	1; 2; 3	[5]
Reference dataset	42.089	4	[15]
EMBL-EBI			
ChEMBL	425.304.329	1; 2; 3	https://www.ebi.ac.uk/chembl/
Chem2Bio2RDF			
BindingDB	20.484	1	https://www.bindingdb.org/bind/index.jsp
Pubchem	78.000.000	2	https://www.ncbi.nlm.nih.gov/pcassay
Bio2RDF			
Drugbank	3.672.531	1	http://www.drugbank.ca/
PubMed	5.005.343.905	4	http://www.ncbi.nlm.nih.gov/pubmed/

To illustrate the remaining basic features of PIBAS FedSPARQL we will introduce the following use case: Researchers from a laboratory have just received a synthesized substance (a drug) and a list of its molecular information from chemists. The information they were provided with includes the molecular formula, molecular weight, InChiKey and SMILES. Before the researchers can determine how they will proceed in their investigation, they are carrying out a pre-screening of the synthesized substance.

**Fig. 4:**
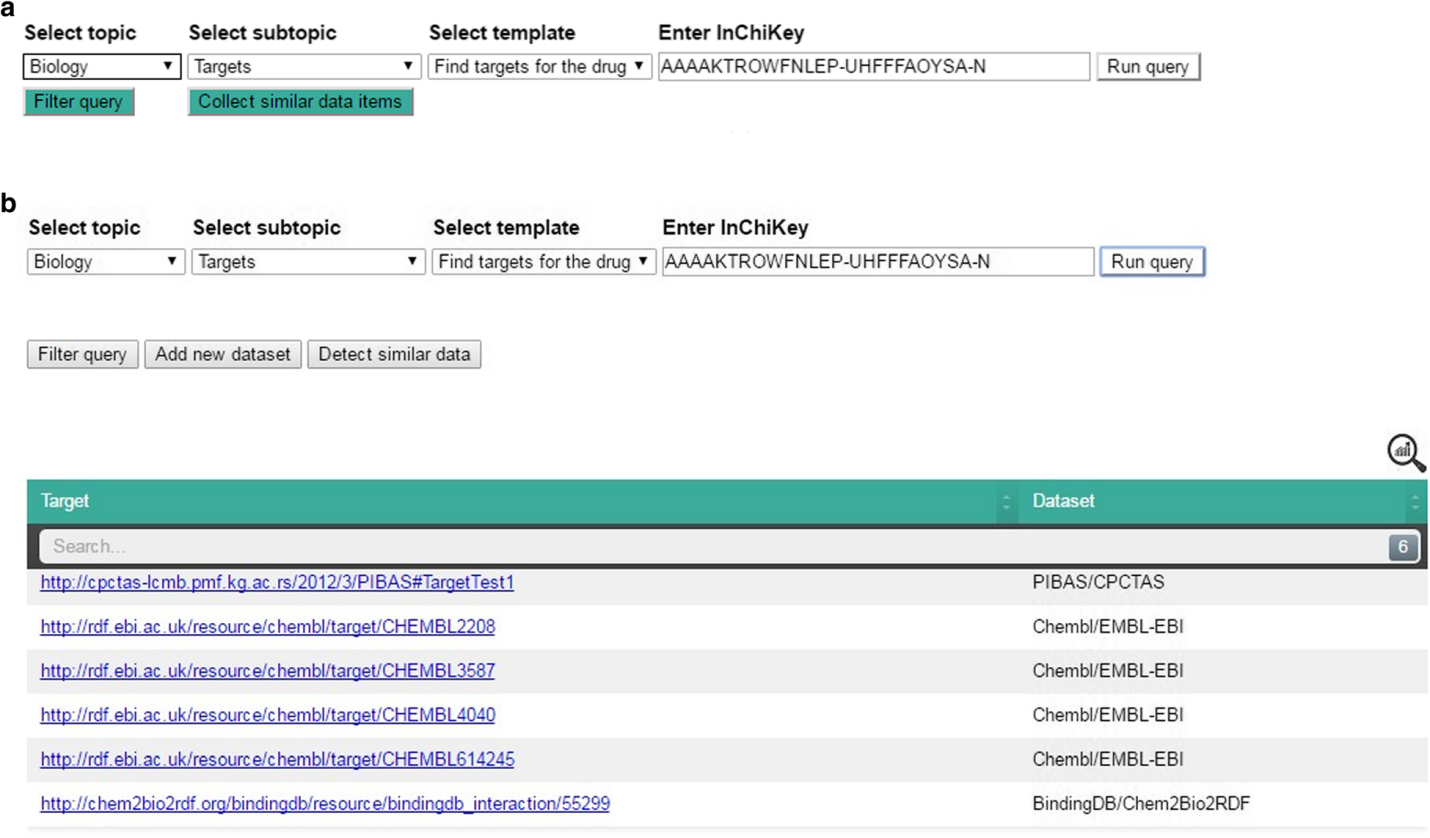
Running of predefined query in PIBAS FedSPARQL **a**) Initial user interface **b**) Results after running predefined query. The initial user interface allows users to create queries in a very simple way by selecting a (sub)topic, template and entering a keyword. By clicking on the “*Run query*” button, the predefined Federated SPARQL query is executed and users receive results in the form of a table. The first column shows the retrieved results as URI or string. The second column displays the data source and initiative name. The icon in the top-right corner of the table shows statistical information about the retrieved data, including data source name, initiative name and the number of obtained data items per data source

The main questions posed in this process are related to whether a substance has already been synthesized and used by other initiatives. Data collected in this way can be useful for further experiments. Suppose that researchers want to find targets for a particular drug that has the following InChiKey: *AAAAKTROWFNLEP-UHFFFAOYSA-N*. After determining the selection criteria and running the query (Fig. 4a) researchers receive information that targets are found in the PIBAS, ChEMBL and BindingDB datasets (Fig. 4b). In this case, the Federated SPARQL query is predefined over four pre-selected datasets, as it is specified in the *DataSources* ontology (see Fig. 2), and it retrieves results from three of them. As in this case, it may happen that endpoints do not contain the requested data or that they are not reachable. Statistical information about the retrieved data can be viewed by clicking the icon in the top-right corner of the results table.

### Adding new datasets

**Fig. 5:**
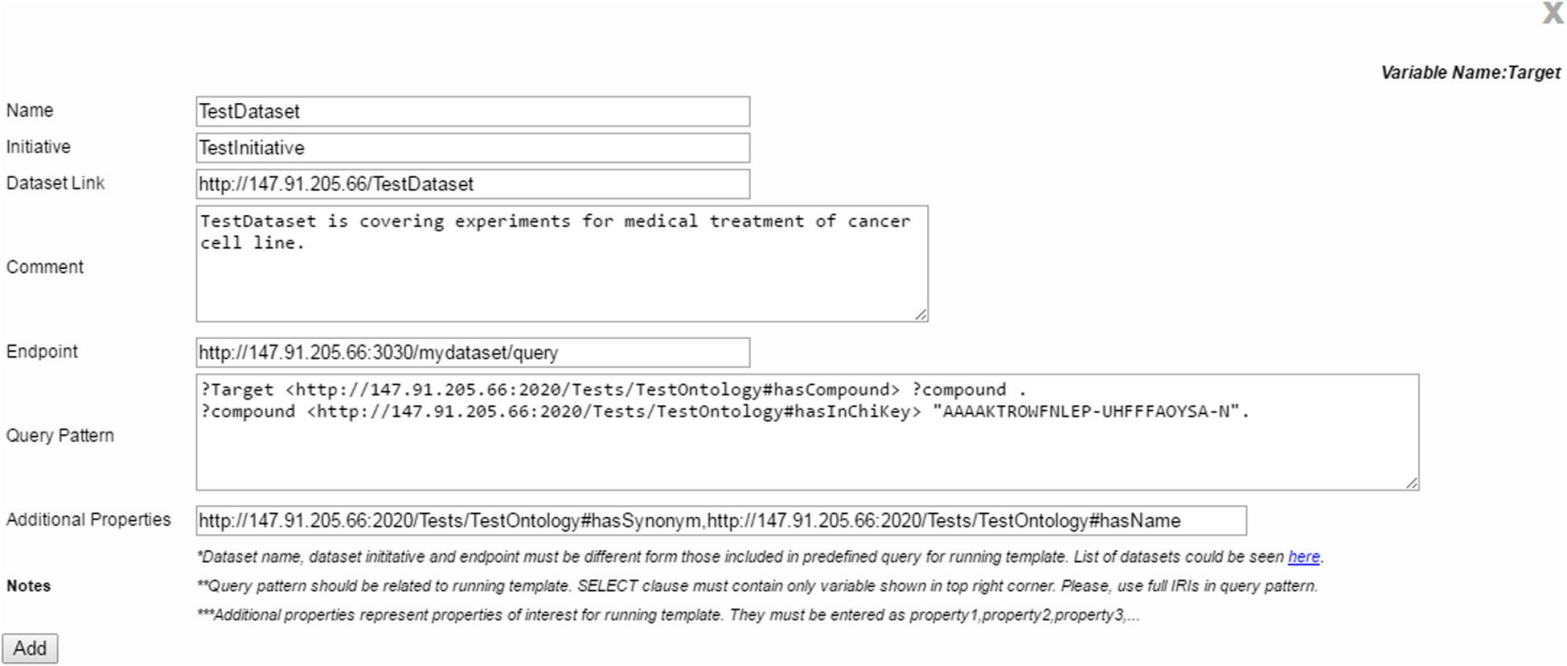
Adding new dataset to predefined query. This figure shows the pop-up window that allows users to incorporate any new dataset not included in the predefined list of datasets for an existing template. Users need to enter the dataset name, initiative name, dataset link, a comment, the endpoint URL, pattern query and the dataset properties most relevant for the selected template and topic. Finally, they need to click the “*Add*” button to complete the action. Conversance with SPARQL and the underlying ontology is necessary for this step

A major issue in bioinformatics research is the sheer volume of information that researchers are faced with. It is often a laborious task to find data relevant or vital to analyzing and interpreting experimental findings in a particular area of research. Data from high-profile projects are usually easily found, but there are also many smaller laboratories. Their data are harder to obtain, but may be related to and complement the research interest at hand. It would therefore be highly beneficial if it were possible to easily explore the datasets of these laboratories. In PIBAS FedSPARQL, all semantically represented data can be integrated and used for further exploring by way of the system’s feature for adding new dataset (Fig. 5).

**Fig. 6:**
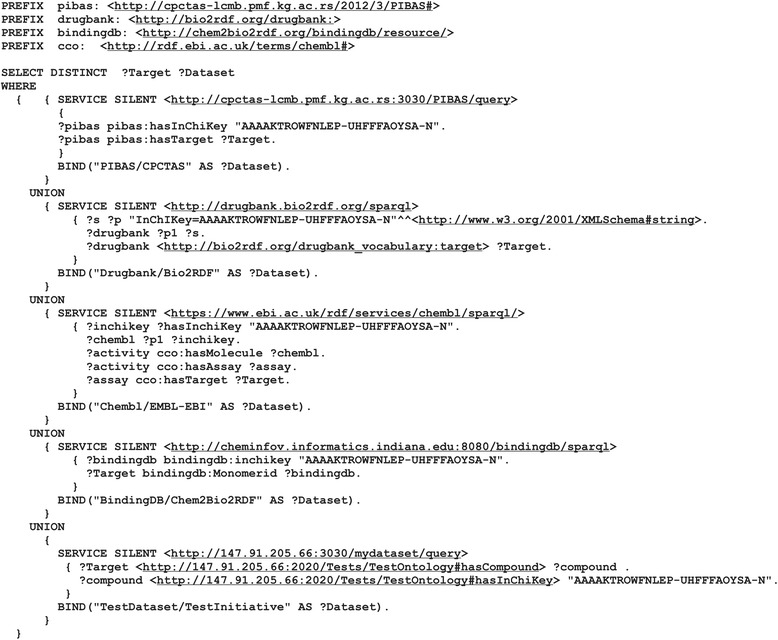
Rewritten predefined query after adding new dataset. This figure shows the rewritten predefined Federated SPARQL query of the template “*Find targets for the drug*” after incorporating a new test dataset

**Fig. 7:**
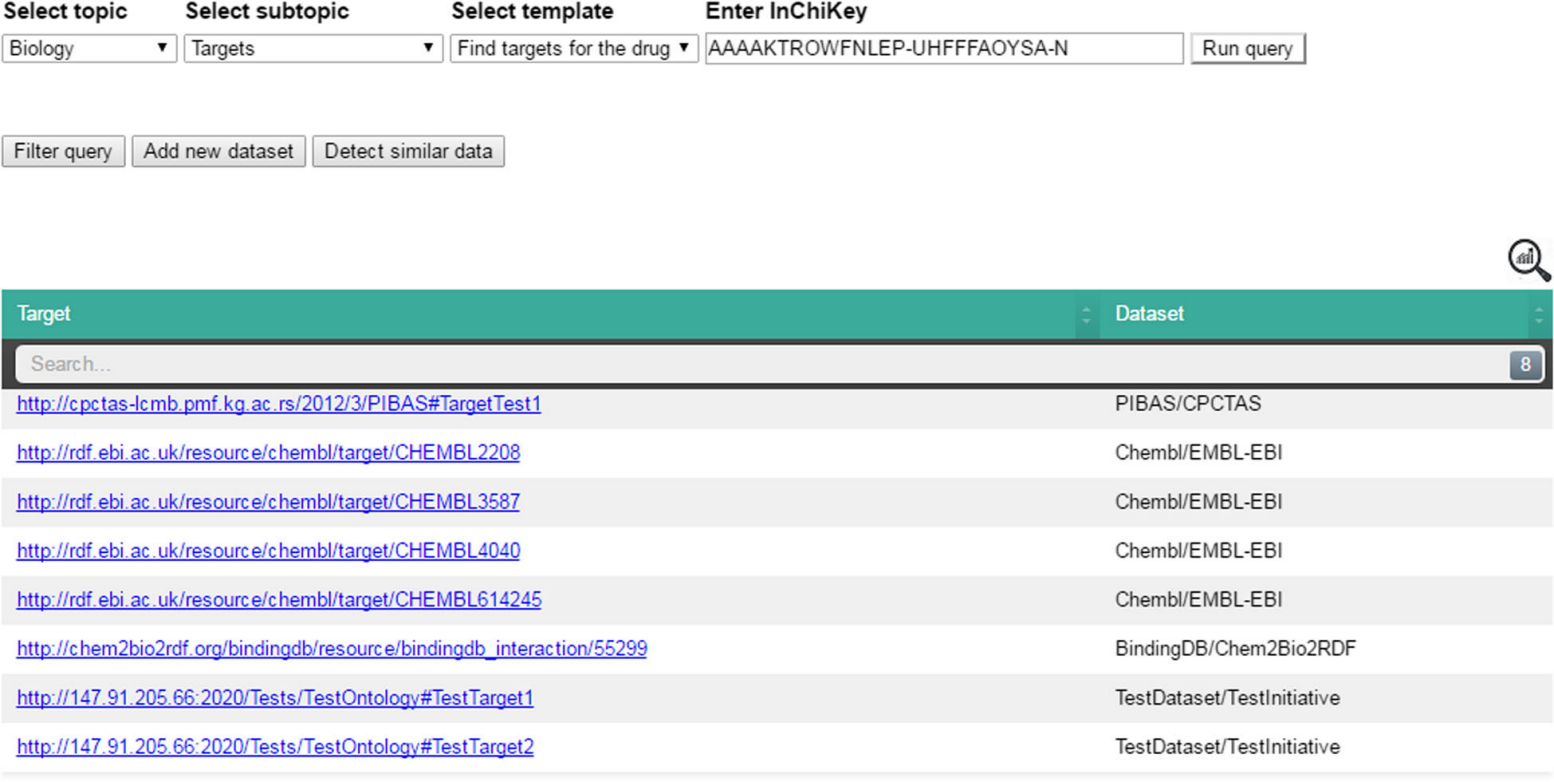
Result set after adding new dataset to predefined query. This figure shows the results in a table after executing the rewritten predefined Federated SPARQL query. The results table has the same layout as in Fig. 4

This feature increases the flexibility of our system and opens the door to a better understanding of data, creating new opportunities for the researchers to perform more productive experiments in the future. By clicking the “Add” button, the researchers can add dataset that is not included in the predefined list of datasets for an existing template. In the pop-up window that appears, the dataset name, initiative name, dataset link, a comment, the endpoint URL, pattern query and some dataset properties that are most important for the selected template and topic have to be entered. The *additional properties* are used for the system’s feature for detecting similar data items. The *pattern query* entered should match a selected topic and template. Following our use case, the pattern query must contain the variable *Target* that matches the name of the running template. The pattern query variable is visible in the top-right corner of the pop-up window for adding new dataset. For testing purposes, we are using a test dataset with a test ontology and a test endpoint.^7^ After entering the basic information, the query preparation component rewrites the original query (Fig. 6) and the researchers can now run a new query. Following this, the rewritten query is evaluated and a more complete answer is returned to the end users (Fig. 7).

### Dynamic query filter

**Fig. 8:**
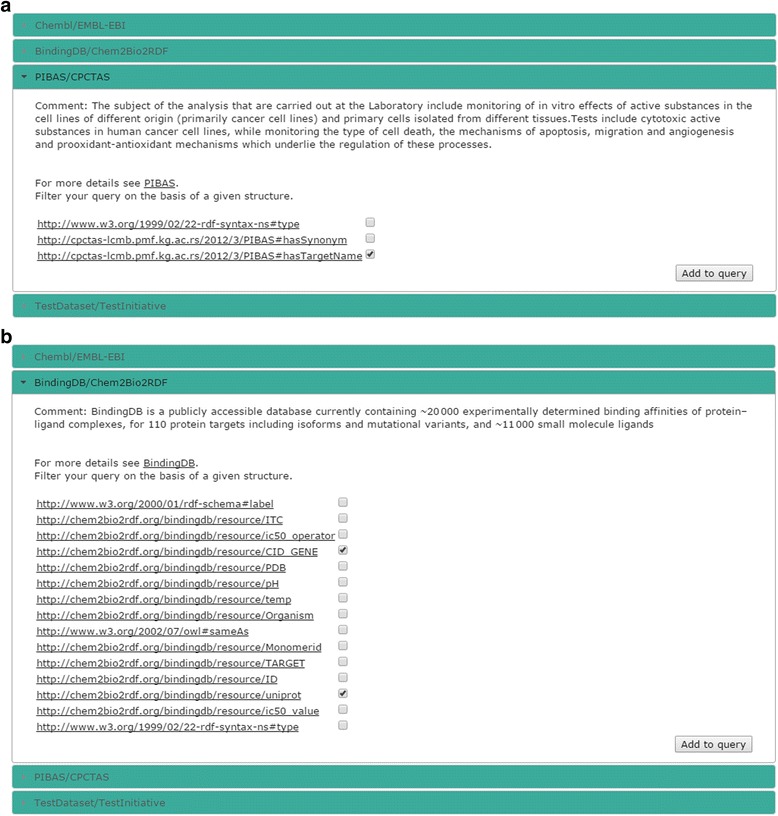
Accordion elements for dynamic query filtering **a**) List of predicates for *PIBAS/CPCTAS* dataset **b**) List of predicates for *BindingDB/Chem2Bio2RDF* dataset. This figure shows the dynamic accordion elements for the *PIBAS/CPCTAS* and *BindingDB/Chem2Bio2RDF* datasets. The accordion elements contain a list of dataset properties which are dynamically created according to the template “*Find targets for the drug*”. Each property listed in an accordion element is hyperlinked to a web page with its description. The same applies to all datasets used in Federated SPARQL query. Users can select their desired properties and add them to the query by clicking on the “*Add to query*” button

The dynamic query filter can be used to obtain additional information. This feature can improve queries by using the underlying structure of datasets without prior knowledge of their structure. By clicking on the “Filter query” button, dynamic accordion elements are created (Fig. 8).

Each dataset used in the query is assigned to an accordion element. Accordion elements are labeled with the dataset name and initiative name. The names are linked, so the end user can directly explore the respective dataset or initiative through their websites or public endpoints. By clicking on an accordion element, it is expanded and automatically populated with the list of properties according to the selected template and topic. This list is generated by running a dynamic SPARQL query in the background. Each property listed in an accordion element has a hyperlink to the web page with its description. This way, end users can analyze properties and determine which of them are relevant for obtaining additional information. Each property can be added to the query by selecting it and the query button “Run query” then changes to “Run new query”.

**Fig. 9:**
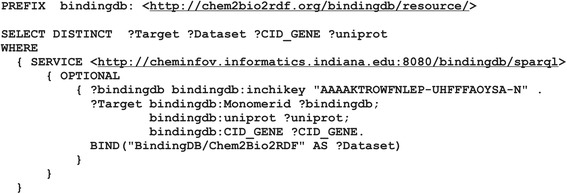
Generated star-shaped query for BindingDB/Chem2Bio2RDF dataset after dynamic query filtering. This figure shows the star-shaped SPARQL query created for the Binding/Chem2Bio2RDF dataset after adding the properties http://chem2bio2rdf.org/bindingdb/resource/CID_GENE and http://chem2bio2rdf.org/bindingdb/resource/uniprot to the query

After the properties selection, a new star-shaped SPARQL query is generated for every dataset of interest. A star-shaped query has one variable as subject and k joins, i.e. (k + 1) triple patterns. Suppose that researchers want to get additional information from datasets used in a predefined query. The focus of their interests could be *http://chem2bio2rdf.org/bindingdb/resource/CID_GENE* and *http://chem2bio2rdf.org/bindingdb/resource/uniprot* properties from the *BindingDB*/*Chem2Bio2RDF* and the property *http://cpctas-lcmb.pmf.kg.ac.rs/2012/3/PIBAS#hasTargetName* from the *PIBAS/CPCTAS* dataset. Figure 9 shows an example of a star-shaped query generated for this particular case.

**Fig. 10:**
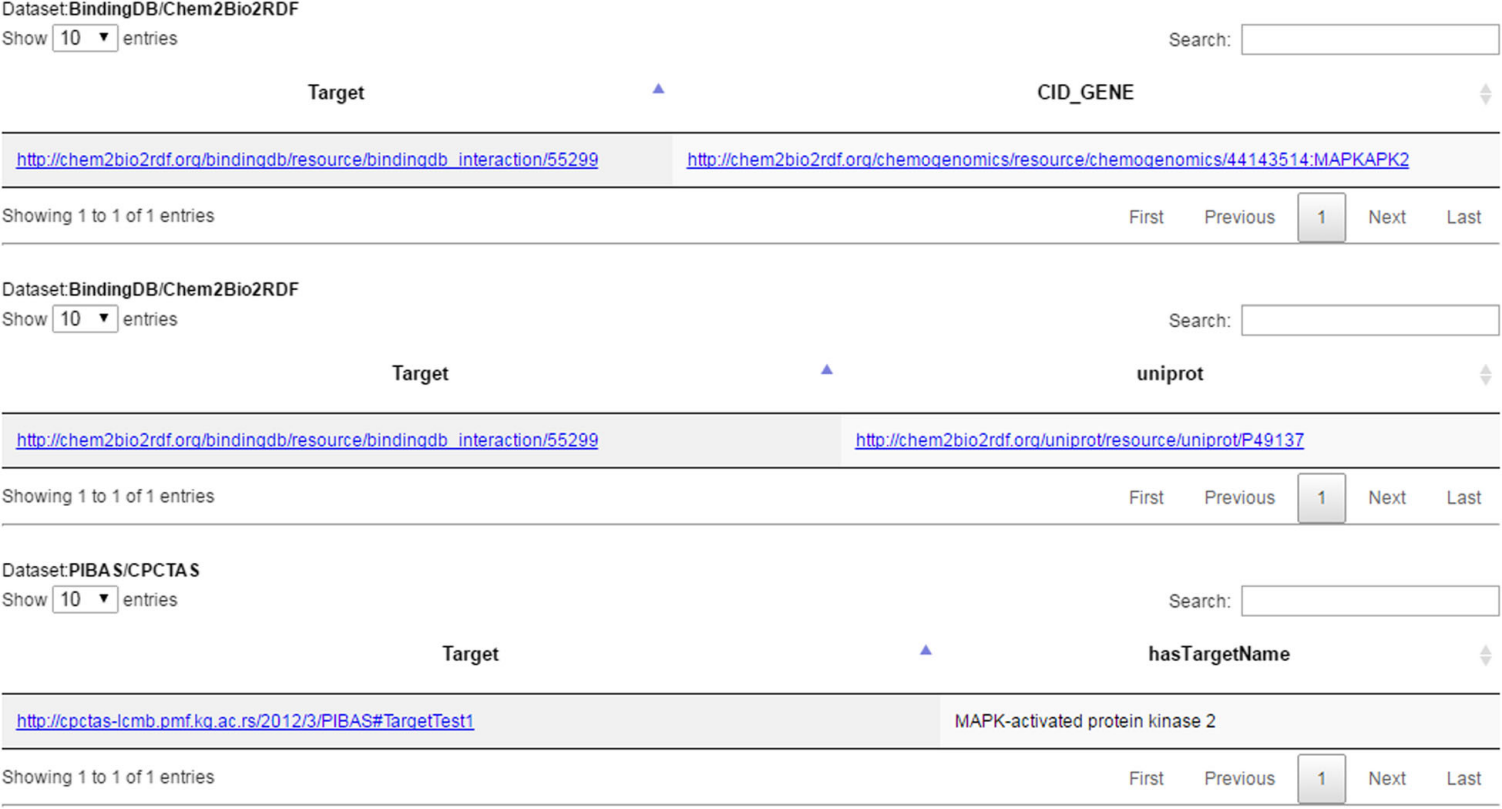
A sample result table after dynamic query filtering. This figure shows the results of dynamic query filtering. The results are organized by source (*PIBAS/CPCTAS* and *BindingDB/Chem2Bio2RDF*) and displayed in a paginated table. They can be sorted and filtered in order to refine the query result and show only the most relevant information

New query results are organized by source and displayed in a paginated table (Fig. 10). Results can be further sorted and filtered in order to refine the query result and show only the most relevant information. This option is particularly useful when dealing with a large number of results.

### Similar data items detection

The development of efficient algorithms for detecting similar data items is an important goal in bioinformatics. The concept of similarity is typical for the study of macromolecular structures, genomes, proteomes and metabolic pathways [51]. Together with the experience and expertise of RC staff, use of similar data items (targets and cell lines) resulted in a greater percentage of successful experiments compared to selecting data items based on intuition. This accelerated the process of obtaining desired results and reduced the cost of performing experiments. In PIBAS FedSPARQL, similar data items detection can be applied to the results of predefined queries as well as to the results retrieved after adding a new dataset. This feature can be manually disabled for some templates in the *DataSources* ontology. Based on the input of RC staff, this option is more important for the *Biology* and *Chemogenomic* topics, than for the *Research* topic for two main reasons. Firstly, finding similar items is more important for topics that will be used for performing further experiments. Secondly, obtained results come in the form of URIs (see Table 4), and our algorithm is applied to URIs, rather than strings. Following the use case specified in this paper, the researchers can find the most similar targets by selecting “Detect similar data items” after adding the test dataset. This is useful as the known targets can be used to make sense of new targets.

As an introduction to a detailed explanation of the algorithm, a brief overview follows: similar data items detection is based on presenting the object values (strings) in the form of a vector and determining the cosines of their mutual angles, which actually represent the degree of their semantic relatedness. The proposed algorithm for similar data items detection is implemented in Python and its pseudocode is presented in Listing 1.
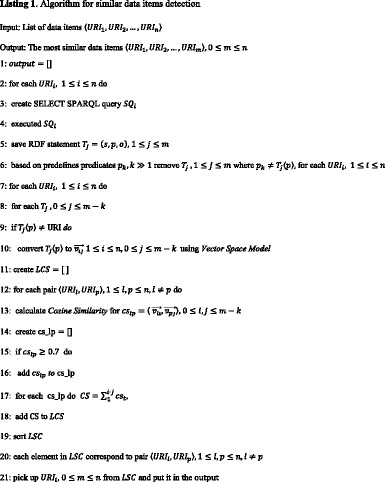


For each data item (URI) in the result set, a SPARQL query that retrieves the entire collection of its predicates and objects is generated and executed (step 3 and 4). Next, based on the RC expert’s decision, the algorithm uses the predicates selected for every template (step 6). Our feature Dynamic query filter assisted RC experts in analyzing and selecting the predicates for every template, i.e. for ontology classes, like *Targets*, *Cell lines* and *Assay* contained in the predefined dataset. Also, when end users add a new dataset to a predefined query, they have to enter the selected predicates (see Fig. 5). Conversance with SPARQL and a profound understanding of the underlying newly added dataset ontology are necessary for this step, because this dataset can be unfamiliar to the bioinformatics community. Generally, the selection of predicates should be performed according to two principles. First, predicates should coincide with the researchers’ interest and the running template. Second, there should be a high occurrence of strings as object values in the RDF statement. For the running template, the selected predicates of the predefined and test dataset can be seen in Table 3. The object values after the selection of predicates can be seen in Table 4.

**Table 3:** Set of selected predicates for running template

Predefined Dataset/Initiative	Selected predicates
PIBAS/CPCTAS	{http://cpctas-lcmb.pmf.kg.ac.rs/2012/3/PIBAS#hasTargetName, http://cpctas-lcmb.pmf.kg.ac.rs/2012/3/PIBAS#hasSynonym}
ChEMBL/EMBL-EBI	{http://www.w3.org/2000/01/rdf-schema#label}
BindingDB/Chem2Bio2RDF	{http://chem2bio2rdf.org/bindingdb/resource/TARGET}
Drugbank/Bio2RDF	{http://purl.org/dc/terms/title, http://www.w3.org/2000/01/rdf-schema#label}
Added Dataset	
TestDataset	{http://147.91.205.66:2020/Tests/TestOntology#hasSynonym, http://147.91.205.66:2020/Tests/TestOntology#hasName}

**Table 4:** Object values after the selection of predicates

Dataset item (URI)	URI abbreviation	Object values after the selection of predicates
http://cpctas-lcmb.pmf.kg.ac.rs/2012/3/PIBAS#TaregtTest1	URI_1_	• MAPKAP kinase 2 • MAPK-activated protein kinase 2
http://rdf.ebi.ac.uk/resource/ChEMBL/target/CHEMBL2208	URI_2_	MAP kinase-activated protein kinase 2
http://rdf.ebi.ac.uk/resource/ChEMBL/target/CHEMBL3587	URI_3_	Dual specificity mitogen-activated protein kinase kinase 1
http://rdf.ebi.ac.uk/resource/ChEMBL/target/CHEMBL4040	URI_4_	MAP kinase ERK2
http://rdf.ebi.ac.uk/resource/ChEMBL/target/CHEMBL614245	URI_5_	THP-1
http://chem2bio2rdf.org/bindingdb/resource/bindingdb_interaction/55299	URI_6_	MAPK-Activated Protein Kinase 2 (MK2)
http://147.91.205.66:2020/Tests/TestOntology#TestTarget1	URI_7_	• MAPKAPK-2 • MAPK-activated protein kinase 2
http://147.91.205.66:2020/Tests/TestOntology#TestTarget2	URI_8_	• Histidine-containing protein • Phosphocarrier protein HPr

**Fig. 11:**
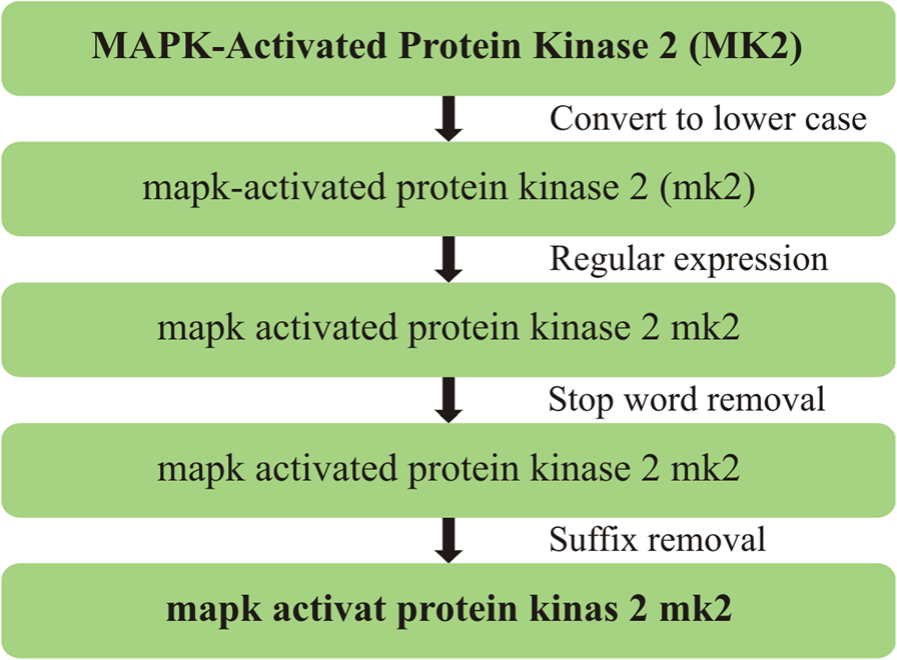
Process of string transformation. The process of string transformation implies conversion and filtering of a string. Initially, the string is converted to lower case. Then it passes through regular expression filtering to extract alphabetic and numeric characters [a-z, 0–9]. The string is then purified by eliminating words that are in the list of stop words. This list contains high-frequency words with relatively low information content (function words and pronouns). Finally, suffix removal is performed by applying Porter‘s Stemming Algorithm [52]

Then, all object values are transformed and prepared to be used in VSM (step 10) to calculate their similarity, as follows: First, all strings are converted to lower case. Then strings are filtered using regular expression to extract alphabetic and numeric characters [a-z, 0–9]. All words from strings are added to a dictionary that keeps track of the words and the number of their occurrences. Before adding a word to the dictionary, a list of stop words is checked that contains high-frequency words with relatively low information content, such as function words (e.g. of, the, and) and pronouns (e.g. them, who, that). For us, it was important to check the stop words before stemming the word, as strings appear to be more related than they really are. In our case, the stemming task (suffix removal) is performed by applying Porter‘s Stemming Algorithm [52]. Figure 11 shows the process of preparing strings to be used in VSM.

**Fig. 12:**
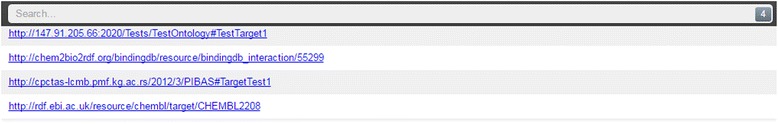
Similar data items (URIs) obtained by our algorithm after adding a new dataset. This figure shows similar targets detected in the results retrieved after adding a new dataset to the “Find targets for the drug” template and running the rewritten predefined Federated SPARQL query. The results are shown in the form of a table on a new web page

In order to achieve the best similarity value between appropriate pairs of vectors we use CSM. CSM achieved better results in comparison with two other Words/n-grams measures, Jaccard Coefficient^8^ and Dice Coefficient,^9^ as shown in [53]. CSM is calculated using formula (1) (step 13). The results from [53] influenced the selection of the threshold, so that pairs of vectors with CSM values below 0.7 are not taken into account. Further, the algorithm sums up the CSM values between each URI pair (step 17). Based on the final sum, similar items for our use case (steps 19–21) are lined up, as represented in Table 5. The final results are shown on a new web page, like in Fig. 12.

**Table 5:** Some running steps in the algorithm for detecting similar data items^*^

Pair of URIs	Pairs of strings/vectors	CMS > 0.7
URI_1_: URI_2_	MAPK-activated protein kinase 2: MAP kinase-activated protein kinase 2	0.800000
URI_1_: URI_6_	MAPK-activated protein kinase 2: MAPK-Activated Protein Kinase 2 (MK2)	0.912871
URI_1_: URI_7_	MAPK-activated protein kinase 2: MAPK-activated protein kinase 2	1.000000
URI_2_:URI_6_	MAP kinase-activated protein kinase 2: Dual specificity mitogen-activated protein kinase kinase 1	0.730297
URI_2_:URI_7_	MAP kinase-activated protein kinase 2: MAPK-activated protein kinase 2	0.800000
URI_6_:URI_7_	MAPK-Activated Protein Kinase 2 (MK2): MAPK-activated protein kinase 2	0.912871
Line up result	{URI_1_-URI_7_: 1.0, URI_1_-URI_6:_ 0,9, URI_6_-URI_7_:0.9, URI_1_- URI_2:_ 0.8, URI_1_-URI_2_:0.8}	
Final result	URI_1_, URI_7_, URI_6,_ URI_2_	

^*^Based on Table 3 and Table 4

## Results

### Evaluation

One of the challenges in the bioinformatics is detection of similar data items across different datasets and initiatives. PIBAS FedSPARQL offers a solution to this problem. In our case, the combination of the VSM and the CSM have a promising role. Evaluation in this context basically means checking if data items are similar. The evaluation task was carried out in cooperation with RC staff, two chemists and five biologists, who participated in the selection process of 29 drug samples. The test set is derived from the experiments at RC, where the cancer cell lines were treated with certain active substances (drugs). Some drug samples are selected arbitrarily. The same RC experts also participated in the selection of the predicates necessary for the evaluation process, during which our Dynamic query filter proved helpful.

The RC experts used drug samples to perform in total 50 queries (test cases): 25 samples on the template 1, 10 samples on the template 2 and 15 samples on the template 3 (some of the samples are used on more than one template). Then, the main evaluation task was applied to the obtained data and the results were analyzed. The analysis process was done manually by RC experts, where the resulting data (URIs) were accessed and compared with each other. The relevant tests cases are presented in Table 6.^10^ Based on human judgment, our algorithm gave accurate results in 92% of test cases, so it can be concluded that our solution is promising for finding similar data items.

**Table 6:** Results obtained from detection of similar data items for different templates

Keyword	Number of data after running predefined query	Number of similar data	Human Judgment
Template label: 1			
MJFJKKXQDNNUJF-UHFFFAOYSA-N	10	8	Yes
MSTNYGQPCMXVAQ-KIYNQFGBSA-N	30	12	Yes
PMATZTZNYRCHOR-IMVLJIQENA-N	5	3	Yes
GUGOEEXESWIERI-UHFFFAOYSA-N	347	99	No
SEKGMJVHSBBHRD-WZHZPDAFSA-M	6	2	Yes
HSMNQINEKMPTIC-UHFFFAOYSA-N	341	106	No
Template label: 2			
C1 = CC(=CC(=C1)F)CSC2 = NC3 = C(N2)C = NC = C3	4	3	Yes
CCOC(=O)C1(CCN(C)CC1)c2ccccc2	104	65	No
C1CCC(CC1)N2C(=O)C3 = CC = CC = C3N = C2SCC#N	3	2	Yes
CC1CCCCC1NC(=S)NC2 = CC = C(C = C2)SC(F)F	2	0	Yes
CC1(CC1(Cl)Cl)C(=O)NNC(=O)C2CCCCC2	7	3	Yes
Nc1nc(O)c2NC(CNc3ccc(cc3)C(=O)N[C@@H](CCC(=O)O)C(=O)O)CNc2n1	23	18	Yes
Template label: 3			
AAAAKTROWFNLEP-UHFFFAOYSA-N	2	0	Yes
MIQPIUSUKVNLNT-UHFFFAOYSA-N	107	41	No
STQGQHZAVUOBTE-VGBVRHCVSA-N	149	50	Yes
UWWDHYUMIORJTA-HSQYWUDLSA-N	2	0	Yes
XCGSFFUVFURLIX-VFGNJEKYSA-N	20	2	Yes
ZPEIMTDSQAKGNT-UHFFFAOYSA-N	117	44	Yes
Total matching: 92% (over all 50 test cases)			

The data obtained from the evaluation process were the basis for further experiments carried out at RC. With the help of our algorithm, it became easier for staff to choose targets and cell lines. It turned out that more similar targets and cell lines provide the same or better results for testing active substances on cancer cells than when they were selected based on intuition. The application of our algorithm has contributed to the publications of some novel results in the field of cancer research [54, 55].

Our evaluation also contains a comparison of two approaches for our algorithm:

- **Predicates selected**: Using user-determined predicates**.**
- **Predicates not selected**: No restriction on predicates.

**Fig. 13:**
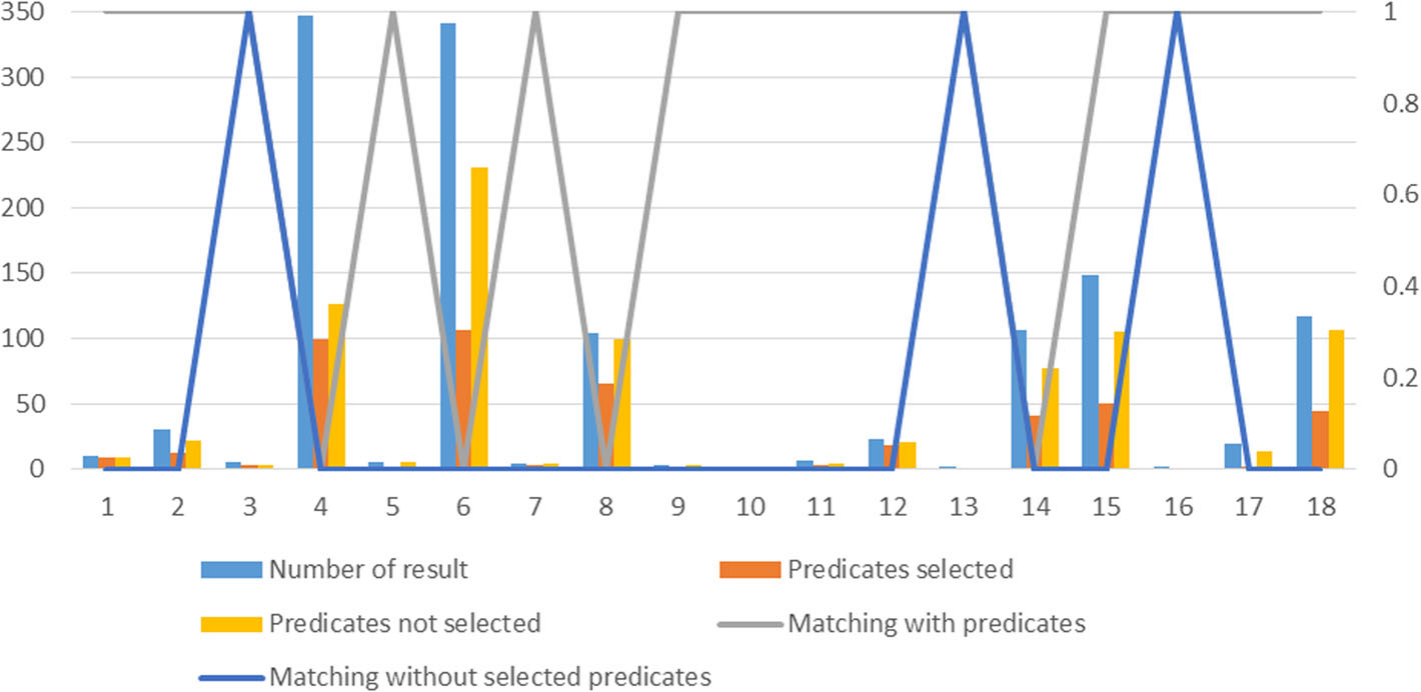
Matching results using methods Predicates selected and Predicates not selected. This figure shows a graphical representation of data from Table 6. The graphic contains the number of similar data items obtained using two approaches, Predicates selected and Predicates not selected, and similarity matching result based on human judgment (0 means that no matching exists, 1 means that a matching exists)

Figure 13 shows the number of retrieved data for every test case from Table 6, the number of similar items obtained using these two approaches and similarity matching based on human judgment (1 means that a matching exist, 0 means that no matching exist). The second approach did not do well in practice. Its score of total matching is only 16.6%. This weaker judgment may be explained by the fact that the algorithm uses non-relevant predicates, which in turn may affect the final results. For example, targets from the *ChEMBL/EBML-EBI* dataset have the property *http://rdf.ebi.ac.uk/terms/chembl#organismName* that represents the source organism of a molecular target or tissue. Many targets could have the same value for this property, for example *Homo sapiens*. Only this successful matching could influence targets similarity, although they are different. Therefore, it is necessary to select predicates. As a consequence, the first approach gives better results.

### Usability and usefulness

**Fig. 14:**
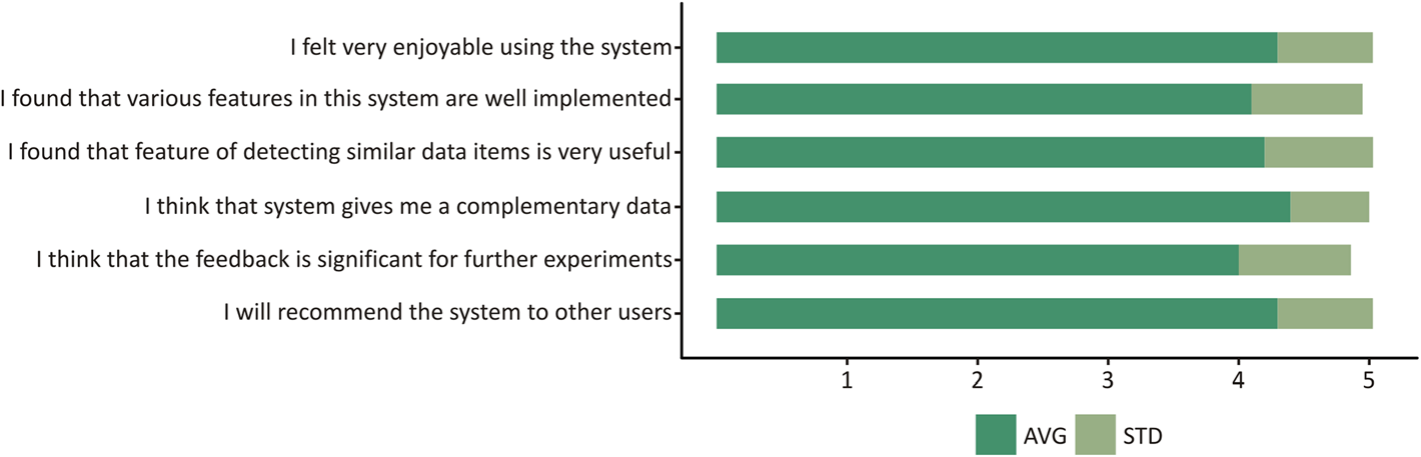
Results of usability evaluation obtained from our questionnaire. This figure shows the final outcome of the survey carried out in cooperation with RC staff. For this survey, the six-item Likert scale-based System Usability (SUS) questionnaire was used. In order to numerically analyze the survey results, the Likert scale responses were translated to numbers using the following five-point scale: 1 = strongly disagree; 2 = disagree, 3 = neutral; 4 = agree; 5 = strongly agree. Based on the questionnaire outcome, average values (AVG) and standard deviation values (STD) were calculated and graphically presented

Cooperating with RC staff during the evaluation process was of great importance because it enabled them to become more familiar with the system. After the evaluation task, through the usage of our system on performing experiments (which produced scientific results [54, 55]), the employees in RC have come to conclusions about the system. We conducted a survey to find out how we could further improve the system according to user requirements. We based the content of the survey on our experience with a similar usability survey for the IMI Python system [56]. The authors used the six-item Likert scale-based System Usability (SUS) questionnaire. In order to numerically analyze the survey results, the Likert scale responses were translated to numbers using the following five point scale: 1 = strongly disagree; 2 = disagree, 3 = neutral; 4 = agree; 5 = strongly agree. The results of the survey are shown in Fig. 14.

The answers to question 1 (4.3 ± 0.73) suggest that our system was very well adopted by end users. The responses to question 2 (4.1 ± 0.85) indicate that the different features left a good impression with the end users. Highly rated question 3 (4.2 ± 0.83) assures us that the end users benefited from our algorithm. This additionally motivated us to continue improving our algorithm in the current direction. The responses to question 4 (4.4 ± 0.6) indicate that our system was helpful for searching for complementary data, that would be used for future experiments. The users positively rated question 5 (average score = 4.0 ± 0.86). This fact implies that our system is a great starting point for finding novel input data used for further experiments. The high rating of question 6 (4.3 ± 0.73) has a positive and encouraging effect on the authors. We will continue to listen to the demands of users and try to tailor the system to their needs. The overall impression of the survey is satisfying and we found the PIBAS FedSPARQL to be very beneficial and useful.

### Limitations

In this section, we outline the known limitations of the system.

Expert knowledge of SPARQL and RDF when adding a new dataset: Proficiency in SPARQL and a profound understanding of the underlying ontology are required as the new dataset could be unknown to the bioinformatics community. In the future work, we may be able to reduce this limitation through integration of our local approach [57]. It would provide easier SPARQL queries management and automatic favorization of predicates.

Endpoint is down: The PIBAS FedSPARQL search relies on the availability of the used remote SPARQL endpoints. Overcoming this limitation by using a local copy of the endpoints is not feasible due to the large size of the data sources measured in terabytes. As a precaution, our system makes use of the ability of Federated SPARQL queries to skip an endpoint which is down with the SILENT keyword.

Duration time of query execution: The initial run of query takes longer than the following iterations, probably due to browser caching. Query execution time may vary significantly according to computer network conditions.

## Conclusion and future work

PIBAS FedSPARQL, is an open-source SPARQL query builder and result set visualizer for bioinformatics data which allows end users to easily construct and run Federated SPARQL queries across multiple datasets. PIBAS FedSPARQL allows users to create queries in a very simple way by selecting a (sub)topic, template and entering a keyword. Currently, (sub)topics and templates are related to the most important requirements of RC staff. All templates provide a great starting point for researchers to find answers to bioinformatics questions. Besides preselected datasets for predefined queries, PIBAS FedSPARQL actively supports end users in adding new datasets for existing queries. After retrieval of the initial result set, query results can be filtered to improve their relevance. Based on projections of individual RDF data sources, queries can be filtered by selecting data which are in the end user’s focus. As an advanced feature, PIBAS FedSPARQL offers the possibility of detecting similar data items based on the given results. We showed that the combination of Vector Space Model and Cosine Similarity Measures offers promising results. Based on end user reviews, we demonstrated that our novel sentence alignment algorithm constitutes an improvement over this baseline. We found that the success of our algorithm mostly depends on suitable predicate selection by experts. In the future, we will focus on automating the favorization of these predicates. We plan to use this strategy to further improve efficacy and usability of our system.

## Availability and requirements

**Project name:** PIBAS FedSPARQL

**Project home page:** http://cpctas-lcmb.pmf.kg.ac.rs/fed/ and https://github.com/marijadjokic/PIBASFedSPARQL

**Operating system(s):** Platform independent

**Programming language:** PHP and Python

**Other requirements:** Modern Browser, i.e. current version of Firefox or Chrome

**License:** GNU GPL

**Any restrictions to use by non-academics:** none

## Appendices

This section contains a short introduction to the basic concepts used in this study. In the following, we use the definitions from the RDF Recommendation [58] and Cosine Similarity for Vector Space Model [59].

**Definition 1** **(URI)**: A Uniform Resource Identifier (URI) is a unique name given to a resource to identify it over a network using specific protocols. URI provides a generic syntax and consists of a generic set of schemes such as URL (Uniform Resource Locator), URN (Uniform Resource Name), URC (Uniform Resource Characteristic), etc. for document (resource) identification.

**Definition 2 (RDF statement)**: An RDF graph *G* is a finite set of RDF statements. For an RDF, the statement is *S* = (*s*, *p*, *o*), where the element *s* is called subject, *p* is called predicate and *o* is called object. A collection of RDF statements can be intuitively understood as a graph: resources, subjects and object are nodes and predicates are arcs connecting the nodes. The set of all values occurring in all triples of *G* (set of URIs and literal values) provides the vocabulary for representing knowledge according to the guidelines for publishing Linked Open Data.

**Definition 3 (Cosine Similarity)**: Cosine similarity is a non-Euclidean distance measure between two vectors. Given two feature vectors $\overrightarrow{c_i}$ and $\overrightarrow{c_j}$ the similarity score between concepts *i* and *j* is represented using the dot product:

1 $$ Sim\left(i,j\right)=\frac{\overrightarrow{c_i}\bullet \overrightarrow{c_j}}{\left|\overrightarrow{c_i}\right|\times \left|\overrightarrow{c_j}\right|} $$

The resulting score is in the range of [0, 1] with 1 as the highest relatedness between concepts *i* and *j*.

## Abbreviations

CPCTAS-LCMB: Center for Preclinical Testing of Bioactive Substances - Laboratory for Cell and Molecular Biology
CSM: Cosine Similarity Measures
PIBAS: Preclinical Investigation of Bioactive Substances
RC: Research Center
RDF: Resource Description Framework
URI: Uniform Resource Identifier
VSM: Vector Space Model

## Footnotes

1
http://cpctas-lcmb.pmf.kg.ac.rs/fed/index.html

2
https://bitbucket.org/openrdf/sesame

3
https://jqueryui.com/accordion/

4
http://graphite.ecs.soton.ac.uk/sparqllib/

5
http://cpctas-lcmb.pmf.kg.ac.rs:2020/

6
http://www.pmf.kg.ac.rs/

7
http://147.91.205.66:3030/

8
https://en.wikipedia.org/wiki/Jaccard_index

9
https://en.wikipedia.org/wiki/S%C3%B8rensen%E2%80%93Dice_coefficient

10 Sometimes, the result of a real-time SPARQL query can contain a lot of data. The results of test cases preformed for this evaluation can be seen at http://cpctas-lcmb.pmf.kg.ac.rs/fed/evaluation/
